# The Evolving Role of Cine MRI in Crohn’s Disease: From Functional Motility Analysis to Precision Management: A Review of the Last 10 Years

**DOI:** 10.3390/diagnostics15233078

**Published:** 2025-12-03

**Authors:** Ali S. Alyami

**Affiliations:** Department of Diagnostic Radiography Technology, College of Nursing and Health Sciences, Jazan University, Jazan 45142, Saudi Arabia; aalmansour@jazanu.edu.sa

**Keywords:** cine MRI, dynamic, CD, diagnosis, treatment

## Abstract

Cine (dynamic) MRI is a non-invasive MRI technique that captures moving images and can be valuable in evaluating inflammatory bowel disease (IBD). This sequence shows emerging potential in providing functional data to assess bowel motility patterns, to aid in the differentiation between predominantly inflammatory (showing reduced peristalsis) and fibrotic strictures (rigid, non-motile segments) and detecting functional obstructions in Crohn’s disease (CD). Unlike static MRI, cine MRI enables clinicians to observe peristaltic movements, aiding in disease characterization and treatment monitoring. Its non-invasive nature and lack of ionizing radiation make it especially useful for repeated assessments in CD. Studies indicate it improves diagnostic accuracy when used with conventional MRI sequences, providing a complementary, functional dimension to the comprehensive management of this chronic condition. While the functional assessment offered by cine MRI presents a significant advantage over conventional static imaging, its clinical translation is currently challenged by high technical variability. Specifically, there is a distinct lack of standardized acquisition protocols (such as field strength, sequence parameters), post-processing software, and universally validated quantitative motility metrics (such as motility index). Therefore, a primary objective of this review is not only to summarize the evolving diagnostic and monitoring applications of cine MRI but also to critically address the methodological inconsistencies and reproducibility hurdles that must be overcome before this technique can be fully integrated into clinical guidelines for precision management of CD.

## 1. Introduction:

Crohn’s disease (CD) is a chronic inflammatory bowel disease (IBD) marked by cycles of relapse and remission [1]. This cyclical process triggers the activation of tissue repair mechanisms, resulting in the accumulation of extracellular matrix components and an increase in mesenchymal cell populations, which include smooth muscle cells, neural hyperplasia, fibroblasts and myofibroblasts [2]. CD can affect any part of the gastrointestinal tract (GI), but it especially affects the terminal ileum, and about one-third of patients present with disease limited to the ileum [3]. Small intestinal motility involves a series of coordinated contractions that produce both retrograde and antegrade peristaltic movements, along with contractions that facilitate mixing within the intestinal lumen [4]. Conditions such as visceral inflammatory neuropathies, irritable bowel syndrome (IBS), functional dyspepsia, constipation, and diabetes can affect small bowel motility. CD can influence the motility of inflamed small bowel segments, a change that might not be visible through fluoroscopy.

Cross-sectional imaging, such as ultrasound and conventional MRI, is a non-invasive technique that provides information on the location, extent, and severity of bowel inflammation in CD [5,6,7,8]. These techniques are also useful for non-invasively monitoring medication treatments. However, these techniques may have limitations in capturing real-time peristaltic activity. Although scintigraphy and intestinal manometry have demonstrated their utility in identifying motor abnormalities in Western countries [9,10], these methods are invasive and can only be conducted at a limited number of facilities [11].

Advanced MRI techniques, such as cine MRI sequence, a type of MRE sequence used to capture motion, have emerged as a non-invasive and promising method for assessing small bowel motility [12,13]. This approach enables the visualization and assessment of GI motility with good temporal resolution, while causing minimal patient discomfort, suggesting low-barrier clinical use and potential for screening and population-based studies [14,15].

Several studies have reported potential clinical applications of cine MRI in CD as well as in functional bowel disorders and GI motility disorders such as IBS [16], functional constipation [17], and chronic intestinal pseudo-obstruction (CIPO) [18], where motility is abnormal and altered compared to healthy subjects. This sequence demonstrates emerging potential in providing functional data to aid in differentiating between predominantly inflammatory strictures, which exhibit reduced peristalsis, and fibrotic strictures, characterized by rigid, non-motile segments. It also helps in detecting functional obstructions in CD. Several studies have investigated the value of cine MRI in CD [19,20]. To the best of the author’s knowledge, no review has been found that explores this topic. Therefore, this review aims to investigate the role of the cine MRI sequence in CD.

## 2. Motility Scan Procedures

Cine imaging techniques generate dynamic images derived from rapid T2-weighted sequences, which can be used to investigate reduced bowel mobility, commonly referred to as the frozen bowel sign. It involves repeatedly acquiring images, typically one per second, at the same anatomical site for the duration of a single breath hold, which usually lasts 15 to 20 s, allowing for the acquisition of small bowel motility [21]. Bowel motility imaging requires careful consideration of various factors, including field strength, fast imaging parameters/sequence, type of oral contrast, scan protocol and post-processing procedures. There is a trade-off between the signal-to-noise ratio (SNR) and temporal and spatial resolutions [22].

Rapid sequences such as balanced steady-state free precession gradient echo and fast imaging with steady-state precession (True FISP) are utilized to image bowel motility while minimizing motion sensitivity quickly. This technique remains reliable even when patients experience breathing difficulties. Due to their high SNR and speed, these sequences are frequently employed in bowel motility imaging. Additionally, image contrast is improved through a combination of T1 and T2 weighting, which enhances fluid visibility and makes the oral contrast solution appear bright on the images, a crucial aspect of bowel imaging [23]. True FISP can be applied for both structural (static anatomical scans) and functional (dynamic motility scans) bowel imaging, achieving higher spatial resolution in anatomical images without compromising temporal and spatial resolutions.

The cine sequence requires no additional patient preparation. Like static imaging, adequate bowel distention with an oral contrast agent is crucial for cine imaging. The oral contrast solution fills the bowel and enhances image brightness. The primary aim of using contrast is to visualize the bowel wall and may also stimulate motility, akin to a cardiac stress test measuring response to a stimulant [24]. A water-based iso-osmotic agent is typically used, providing a high signal on T2-weighted images and a low signal on T1-weighted images. Before the scan, the adult patient begins ingesting the contrast 45–60 min before with a total volume of 1000–1500 mL [24,25,26]. To ensure even and adequate distention of the entire small intestine, administer 200 mL every five minutes. The dosage and method can be adjusted appropriately for special populations such as the elderly, children, and pregnant women.

This technique is typically obtained in the coronal plane, covering the entire abdomen. When diagnosing IBD, including the terminal ileum in the field of view is critical. Patients can be positioned either prone or supine, depending on the institution. The prone position is more preferable because it minimizes motion artifact and abdominal thickness. Sagittal and axial planes can also be performed. Multi-slice and multiphase formats can be obtained in the cine sequence. Multiple phases are captured at each slice location in the coronal plane, from anterior to posterior, to cover almost the entire abdomen. Cine images must be obtained before administering any antiperistaltic drug such as glucagon or N-hyoscine (BuscopanÒ, Boehringer Ingelheim, Basel, Switzerland), if such an agent is used for the remainder of the study. This is necessary to avoid paralyzing the bowel before motility evaluation. Cine MRI has some advantages and disadvantages in assessing the bowel wall in IBD in general, see Table 1.

The use of cine MRI in pediatric gastroenterology, especially for inflammatory bowel disease (IBD), offers significant opportunities beyond just feasibility. Several clinical factors should be considered, such as sedation needs, free-breathing protocols, shorter acquisition times, age-specific normal ranges, and physiological considerations essential for its effective and meaningful use. Motion artifacts are one of the main challenges in pediatric MRI, particularly because of the length of motility sequences. While traditional MRI often requires sedation for anxious or younger children to maintain image quality, cine sequences—which are fast-acquisition sequences like balanced steady-state free precession (bSSFP)—are naturally more resistant to motion. This is driving efforts to eliminate or reduce sedation entirely. Another way to reduce motion artifact is by using advanced reconstruction, such as parallel imaging and compressed sensing, which allow for highly undersampled data acquisition (meaning less time is spent scanning), combined with sophisticated motion correction during image reconstruction.

## 3. Methods

### 3.1. Databases and Search Strategy

A comprehensive literature search was conducted to identify relevant studies that utilized cine MRI for the diagnosis and management of IBD. Multiple databases, including PubMed and Ovid, were used for this review. The search strategy utilized Medical Subject Headings (MeSH) with relevant keywords, such as Cine MRI OR dynamic MRI OR Motility MRI OR functional MRI OR MR enterography. AND (“Crohn’s disease” OR CD) were employed.

### 3.2. Eligibility Criteria

Review articles, editorials, case reports, non-English publications, and abstract-only articles were excluded. Moreover, studies that were not directly relevant to the topic, as well as those for which the full text was inaccessible, were excluded from consideration. The review focused on other studies, such as clinical trials, observational studies, published over the last decade (2015–2025). Moreover, studies conducted in adults and pediatric populations were included. However, studies before 2015, included in the introduction, are used to explain the basics of cine MRI or the background of IBD. Reference lists of all identified relevant review articles were manually screened to identify additional pertinent studies. Data extraction was conducted independently by the primary author, with a focus on study characteristics (including authors and design), patient demographics, scanner strength, reference standards utilized, and key findings related to the application of cine MRI or motility assessments in patients with CD. Due to the heterogeneity and scarcity of the literature on this topic, as well as the scope of the narrative review format, no quantitative synthesis or bias assessment was performed.

## 4. Using Cine MRI in CD Motility in Adults and Pediatric

### 4.1. In Adults

**Figure 1:**
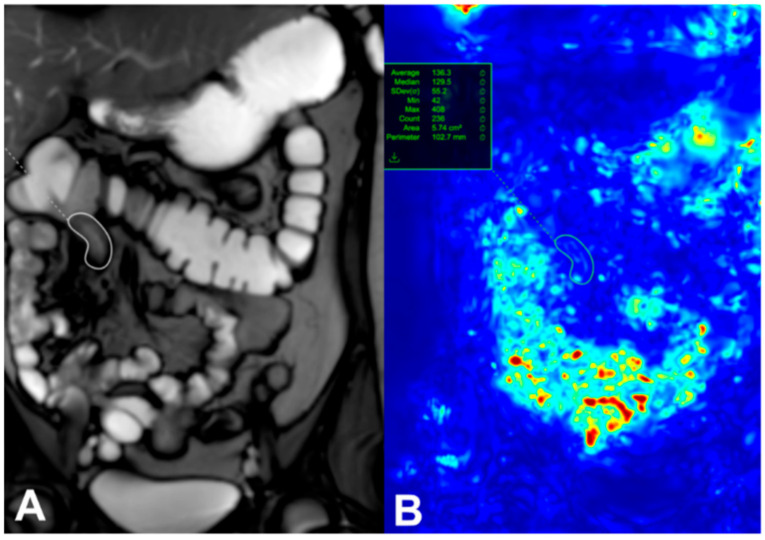
A single image from a dynamic set with a region of interest (white circle) placed on a diseased terminal Ileum (**A**). Motion is assessed by assigning each pixel in the image an associated displacement value and is expressed as a quantitative value (green circle) (**B**) [8]. This figure was taken from [8].

Patients with active CD are known to have reduced intestinal motility, which can be measured using cine sequences. The mechanisms behind decreased motility in CD-affected bowel are multifactorial, involving fibrotic and inflammatory infiltration, neuritis within the bowel walls, and systemic effects of inflammation through neuronal and hormonal pathways. Advances in MRI technology now allow for the capture of small-bowel motility in a single breath hold, and post-processing software can quantify this motility, as shown in Figure 1 [8]. This figure was taken from Figure 6 of Rimola et al.’s study [8].

Previous work investigating cine MRI has concentrated largely on its association with disease activity for assessing CD strictures [27]. In a retrospective study of 59 CD patients with small bowel lesions, Lambrou et al. [27] investigated the association between cine MRI motility abnormalities and complications in CD, focusing on strictures and penetrating disease. The study reported that qualitative cine MRI analysis revealed that decreased motility correlated not only with inflammatory markers (hyperenhancement, comb sign; *p* < 0.05) but also with strictures (OR 0.40, *p* = 0.038), independent of inflammation. In particular, 90% of cases showed reduced or absent motility, while fistulas were not significantly associated with motility changes. The study emphasizes the usefulness of cine MRI in detecting strictures, a key feature of fibrotic complications, which complements conventional MRE sequences.

In a retrospective study, Hahnemann et al. [28] in 2015 introduced a quantitative MRI-based method for assessing small bowel motility in patients with 30 CD. Using dynamic 2D RARE MRI sequences at 1.5 T, the researchers applied an optical flow algorithm to generate parametric motility maps (mean vector maps and mean motility maps) that objectively quantify bowel movement. Both the entire gastrointestinal tract and the inflamed segments were analyzed. The study reported the mean motility score in inflammatory regions, which showed significantly reduced motility (mean 1080 vs. 2839 in the whole GI tract, *p* < 0.0001). Compared to the mean motility maps of the whole GI tract, the inflamed segments showed a decrease in motility ranging between 20% and 87%. Moreover, the inflamed segments correlated strongly with established MRI activity scores (r = −0.59, *p* = 0.0003) and lesion length (r = −0.35, *p* = 0.046), suggesting that motility quantification could serve as an additional biomarker for inflammation activity.

Several studies have examined the association between histopathologic and endoscopic CD activity with reduced motility [20,29] and motility recovery, which may be a useful marker for treatment response [30,31,32]. A prospective study [29] aimed to investigate the relationship between MRI-quantified small bowel motility with inflammation (fecal calprotectin, fC) and Harvey–Bradshaw Index (HBI) in 53 CD patients. The study reported that motility variance was significantly negatively correlated with total HBI (rho = 20.45, *p* = 0.001), fC (rho = 20.33, *p* = 0.015). However, there was no correlation between mean motility and fCor HBI (*p* > 0.05). The authors concluded that reduced motility variance in morphologically normal small bowel is associated with fC levels and patient symptoms.

A multicenter prospective study conducted by Menys et al. [20] involved 82 patients with CD who underwent MRE and colonoscopy within a two-week interval. The study aimed to investigate the use of MRI-quantified small bowel motility as a biomarker for CD activity, comparing it against histopathologic and endoscopic reference standards. The study findings revealed that reduced motility strongly correlated with both endoscopic and histopathologic markers of inflammation. Specifically, motility scores showed high sensitivity (Sn.) (93% for Crohn’s Disease Endoscopic Index of Severity (CDEIS) and 92% for Endoscopic biopsy Assessment of Inflammatory Activity (eAIS) in detecting active disease, though specificity (Sp) was more modest (61% and 71%, respectively). When compared to the MR Index of Activity (MaRIA), a structural MRI-based scoring system, motility performed similarly or better in Sn but with slightly lower Sp. Receiver operating characteristic analysis revealed no significant differences in diagnostic performance between motility and MaRIA, suggesting both methods are viable for assessing CD activity. They concluded that reductions in segmental bowel motility are inversely correlated with endoscopic and histopathological activity grades, and recovery of motility may better capture early treatment response than morphological measurement.

A recent investigation by Dreja et al. [33] examined changes in small bowel motility in individuals with active CD compared to HCs. The study included 134 CD patients and 22 HCs with motility index measured using a semi-automated tool (GIQuant). This study introduced a novel approach by assessing motility not only in the terminal ileum, the primary focus of most previous research, but also in the jejunum and other ileal segments. The findings revealed that motility disturbances were confined to areas of active inflammation, with no significant impact on non-inflamed regions. In HCs, the motility patterns observed in the terminal ileum were consistent throughout the entire ileum, whereas the jejunum exhibited distinct characteristics. Additionally, their findings indicated an inverse relationship between motility index and mural thickness in the terminal ileum. Patients with active CD exhibited a lower motility index compared to the HC in the ileal region.

Beek et al. [34] conducted a prospective cross-sectional study measuring motility on cine MRI in 28 adult patients with CD to determine if it can distinguish chronic (i.e., non-inflammatory) strictures from inflammatory (including inflammatory and mixed) strictures. The GIQuant method analyzed small bowel motility before strictures and during dilation of strictures. Among the participants, 15 pre-strictures and 30 strictures during dilation were identified. Chronic (non-inflammatory) strictures showed higher pre-dilation motility than inflammatory (including mixed) strictures (289.5 AU [188.0–362.9] vs. 113.1 AU [83.6–142.4], *p* = 0.004). The AUC for detecting chronic (non-inflammatory) strictures was 0.93 (95% CI 0.78–1.0, *p* = 0.01). Within the strictures, motility did not significantly differ across histopathology categories (*p* = 0.6).

In a recent study published in 2025, van Harten et al. [35] investigated an innovative method for quantifying small intestinal motility using 3D cine MRI with centerline-aware motion estimation. They developed an automated process for tracking and characterizing intestinal motion patterns in 3D by combining neural networks with deformable image registration. Their method measures local velocities along intestinal centerlines while removing motion artifacts caused by adjacent or breathing structures. The study included 10 patients with CD (87 small intestinal segments) and 14 HCs (127 small intestinal segments). They found that the model achieved a strong performance in differentiating between motile and non-motile segments (AUC 0.97) and in identifying peristaltic motion (AUC 0.81). The absolute velocity (which reflects the magnitude of motility) of intestinal content differed significantly between CDs and HCs (median [IQR] 1.06 [0.61, 1.56] mm/s vs. 1.84 [1.37, 2.43] mm/s), consistent with manual annotations of motile activity. The results revealed significantly reduced motility in CD (median velocity 1.06 mm/s) compared to HCs (1.84 mm/s).

### 4.2. In Pediatrics

Different factors should be considered during pediatric examination, as mentioned in the previous section. In pediatric CD patients, limited data discuss the role of cine MRI in children with CD. Similarly to adults, reduced motility was observed in pediatric patients. A recent retrospective study demonstrated that quantitative MRI could evaluate bowel motility in 25 pediatric patients with CD in the United Kingdom (UK). Coronal free-breathing bSSFP with a temporal resolution of approximately 271 ms for the whole abdomen. It showed that as the extent of inflammation increases, bowel motility decreases. The study reported no significant relationship between fC and quantified intestinal motility (r = −0.27, *p* = 0.40). However, they reported that a correlation was found between motility and eAIS (r = −0.58, *p* = 0.004, *N* = 23) and also between motility and CDMI (r = −0.42, *p* = 0.037, *N* = 25). The motility score was lower in active disease (median 0.12 vs. 0.21, *p* = 0.020) while CDMI was higher (median 5 vs. 1, *p* = 0.04). Terminal ileum motility was negatively associated with CD activity. The study also indicated that a reproducible and valid motility score could allow existing MRE protocols to be shortened and performed with a free-breathing method. This would be highly beneficial for younger children with CD, as they are often unable to remain still for extended periods and have more difficulty cooperating with breath-holding techniques currently used in MRE protocols [36].

A recent single-center retrospective study by Meshaka et al. [37] aimed to investigate inter-reader agreement among radiologists of different experience levels and assess the clinical utility of the motility index as a biomarker for disease response. The study included 64 pediatric IBD cases: 3/64 (5%) UC; 57/64 (89%) CD; 2/64 (3%) very early onset IBD; and 2/64 (3%) IBD unclassified patients with ≥2 MREs, including cine MRE. The GIQuant^®^ software calculated the motility index (arbitrary units, a.u.s) via pixel-wise Jacobian standard deviation. Coronal free-breathing bSSFP with a temporal resolution of 30 frames/8 s/slice position using 1.5 T or 3 T MRI scanners was used in this study. The study demonstrated good-to-excellent reproducibility in quantified bowel motility assessment across radiologists of varying expertise. Junior readers (trainees) achieved 73–84% agreement with experienced readers in identifying the direction of motility change (improvement or deterioration), while senior readers (early-career consultants) showed 67–85% agreement. Bland–Altman analysis revealed no systematic bias and clinically acceptable limits of agreement (±138 arbitrary units) for change motility index measurements. There was no significant difference in Se, Sp, or percentage agreement with the reference standard by clinical gastroenterology or experienced radiologists with the addition of the motility index. Integrating the motility index into MRE interpretation did not enhance diagnostic accuracy for identifying treatment response.

A recent retrospective study included 50 pediatric patients with ileal or cecal CD, all of whom underwent MRE and endoscopy within seven days without intervening treatment, to investigate the utility of quantitative MRI-based motility assessment [38]. The researchers derived motility scores using the GIQuant software to analyze cine MRI sequences. Coronal free-breathing bSSFP with a temporal resolution of 15 s/slice using 1.5 T and 3 T MRI scanners was performed. The findings reported a strong inverse correlation between the MRE-based MaRIA score and motility scores (ρ = −0.66, *p* < 0.0001), confirming that decreased bowel motility correlates with increased inflammation. While the MaRIA score also showed moderate correlations with CDEIS and histological (eAIS) indices, the direct relationship between motility scores and these endoscopic measures was not statistically significant. An overview of clinical studies utilizing cine MRI for adult and pediatric CD is provided in Table 2.

## 5. Using Cine MRI in CD Treatment in Adults and Pediatric Patients

### 5.1. In Adults

Cine MRI is an intuitively appealing sequence for predicting therapeutic response because it measures gut function (such as peristaltic activity). As part of the standard MRE protocols, bowel wall motility could be a reproducible treatment biomarker. In a mixed retrospective/prospective study of 46 patients receiving anti-TNFα therapy, Plumb et al. [31] demonstrated that MRI-quantified small bowel motility is a sensitive marker of therapeutic response in CD. The study reported that responders showed a median increase of 73.4% in motility, while non-responders experienced a 25% reduction (*p* < 0.001). Motility changes correlated strongly with clinical response (93.1% Sn, 76.5% Sp), MaRIA score improvements (*p* = 0.017), and CRP normalization (*p* = 0.035). Notably, motility improvements were detectable as early as 12 weeks post-treatment, suggesting utility for early response assessment.

A prospective study included 15 controls and 20 children and young adults with newly diagnosed ileal CD [30]. It assessed motility changes at baseline, 6 weeks, and 6 months post-treatment using cine MRI and FDA-approved software (GIQuant). The study did not compare histologic and endoscopic scores. Cine imaging was performed at six to eight slice locations, involving the terminal ileum, using a coronal 2D balanced steady-state free precession sequence. The study findings showed that intestinal motility scores increased significantly after initiating biologic therapy, with improvements observed as early as 6 weeks. However, no significant difference was found between baseline motility scores in CD patients and controls, possibly due to varying disease activity. A novel normalized motility score (comparing the terminal ileum to the non-inflamed proximal ileum) better distinguished CD patients from controls (AUC = 0.88) and correlated more strongly with treatment response. The study highlighted excellent interobserver agreement (ICC = 0.89) but noted weak correlations between motility scores and traditional biomarkers such as fC. Moreover, when conventional terminal ileal intestinal motility scores were used, there was no significant difference in conventional intestinal motility scores between CD patients and control participants. Additionally, this study demonstrates a correlation between intestinal motility scores and several clinical indicators of inflammatory burden, such as CRP level, the erythrocyte sedimentation rate and weighted Pediatric Crohn Disease Activity Index (wPCDAI). The results suggest that cine MRI assessment could serve as an objective, non-invasive tool for monitoring early treatment response in CD. However, further research is needed to confirm its clinical relevance and utility.

A recent prospective, multicenter trial (Motility Study) involved 86 patients with active non-stricturing CD before starting anti-TNFα or anti-IL-12/23 therapy and post-induction therapy (visit 2: 12–30 weeks) [32]. The trial investigated whether small bowel motility measured by cine MRI could predict long-term response to biological therapy in CD patients, compared to traditional biomarkers such as fC and C-reactive protein (CRP). The response was defined as a ≥50% drop in either the London Index or simple endoscopic score-CD (SES-CD) between visits 1 and 3. They found that stable or improved cine MRI post-induction therapy was more sensitive than CRP normalization for predicting response or remission (71% vs. 45.2%) but less specific (30.9% vs. 67.3%). The AUC of the ROC for the two tests was not significantly different (*p* = 0.65), at 0.53 for CRP and 0.48 for MRI. Similarly to comparison with CRP, cine MRI was considerably more sensitive than normalization of fC to predict response or remission at 1 year (64.4% vs. 7.1%) but less specific (31% vs. 79.3%). The ROC of the AUC for two tests was not significantly different (*p* = 0.41), at MRI 0.58 and fC 0.67. Neither cine MRI, CRP, nor fC reliably predicted quality-of-life improvements at one year. The study concluded that while cine MRI is a useful marker of active inflammation, it lacks prognostic utility for long-term treatment outcomes. The findings suggest that early changes in cine MRI, CRP, or fC should not guide long-term therapeutic decisions in CD. Videos S1 and S2 in the Supplementary File show the bowel motility of a CD patient before and after treatment. These videos are courtesy of MOTILITY trail raw data.

As part of the MOTILITY Trial, Hameed et al. [39] evaluated the reproducibility of MRI-based small bowel motility measurements (cine MRI) in CD patients, focusing on inter- and intra-observer variability. They involved 297 segmental small bowel motility scores from 104 patients with active non-stricturing small bowel CD, with motility assessed using GIQuant software by both experienced and inexperienced radiologists. The study findings reported that moderate inter-observer agreement for cine MRI measurements, with ICC of 0.59 (95% CI: 0.51, 0.66) for experienced readers and 0.70 (95% CI: 0.61, 0.78) for inexperienced readers. Intra-observer agreement was also moderate (ICC 0.70–0.71) for the two cine MRI-experienced radiologists. Bland–Altman analysis showed tighter agreement for less mobile (diseased) bowel segments, while variability increased with higher motility scores (reflecting normal or responding bowel). An overview of clinical studies utilizing cine MRI for adult CD is provided in Table 3.

### 5.2. In Pediatrics

Cine MRI is particularly well-suited for the pediatric population due to its entirely non-invasive nature and absence of ionizing radiation, making it ideal for repeat assessments. Studies in pediatric CD have largely mirrored adult findings, confirming that reduced bowel motility correlates significantly with active inflammation. A retrospective study demonstrated that quantitative MRI could evaluate bowel motility in 25 pediatric patients with CD in the United Kingdom (UK). It showed that as the extent of inflammation increases, bowel motility decreases. The study reported no significant relationship between fC and quantified intestinal motility (r = −0.27, *p* = 0.40). Terminal ileum motility was negatively associated with CD activity. The study also indicated that a reproducible and valid motility score could allow existing MRE protocols to be shortened and performed with a free-breathing method. This would be highly beneficial for younger children with CD, as they are often unable to remain still for extended periods and have more difficulty cooperating with breath-holding techniques currently used in MRE protocols [36].

A recent single-center retrospective study by Meshaka et al. [37] aimed to investigate inter-reader agreement among radiologists of different experience levels and assess the clinical utility of the motility index as a biomarker for disease response. The study included 64 pediatric IBD 3/64 (5%) UC; 57/64 (89%) CD; 2/64 (3%) very early onset IBD; and 2/64 (3%) IBD unclassified patients with ≥2 MREs, including cine MRE. GIQuant^®^ software calculated the motility index (arbitrary units, a.u.s) via pixel-wise Jacobian standard deviation. The study demonstrated good-to-excellent reproducibility in quantified bowel motility assessment across radiologists of varying expertise. Junior readers (trainees) achieved 73–84% agreement with experienced readers in identifying the direction of motility change (improvement or deterioration), while senior readers (early-career consultants) showed 67–85% agreement. Bland–Altman analysis revealed no systematic bias and clinically acceptable limits of agreement (±138 arbitrary units) for change motility index measurements. There was no significant difference in Sn, Sp, or percentage agreement with the reference standard by clinical gastroenterology or experienced radiologists with the addition of the motility index. Integrating the motility index into MRE interpretation did not enhance diagnostic accuracy for identifying treatment response.

In a recent retrospective study aimed to characterize ileal strictures in pediatric and young adult patients (age 10–28 years) with CD who were receiving biologic therapy, by exploring the role of bowel wall motility in predicting therapeutic response compared to standard MRE biomarkers [40]. The group included 40 participants with imaging-confirmed ileal strictures (defined by CONSTRICT criteria) who underwent escalation or modification of their biologic therapy. Clinical response was assessed up to 6 months after treatment adjustment, classifying patients as responders (*N* = 25) or non-responders (*N* = 15). Non-response was defined as increased disease activity, the need for surgical intervention, or discontinuation of therapy. Cine MRI was acquired using a coronal plane with a 2D bSSFP sequence at 1.5 T and 3 T. The study found that traditional MRE biomarkers such as age, sex, stricture length, wall thickness, stricture volume, T2-weighted signal intensity, and the sMaRIA score showed no statistically significant differences between responders and non-responders. However, there was a significant difference in mean stricture motility between the groups. Responders had significantly higher mean motility (176.03 ± 128.63 AU) compared to non-responders (67.83 ± 33.88 AU, *p* = 0.006). Multivariable analysis confirmed that higher mean motility was associated with increased odds of being a responder (OR: 1.020; 95% CI, 1.00–1.04; *p* = 0.04). Additionally, the Motility score outperformed sMaRIA in predicting non-response, with a higher AUC of 0.81 for motility versus 0.61 for sMaRIA overall. A subgroup analysis of patients aged 21 years or younger showed similar results, with motility achieving an AUC of 0.75 compared to 0.61 for sMaRIA. The study concluded that quantitative MRI motility assessment is a superior predictor of therapeutic response to biologic therapy than traditional MRI biomarkers of inflammatory activity in adolescents and young adults with stricturing CD [40]. Table 4 summarizes some studies that utilize cine MRI in the treatment of pediatric CD.

## 6. Role of Cine MRI in Differentiating Mural Fibrosis and Active Disease in CD

The differentiation of active inflammation from fibrosis in CD remains a persistent clinical challenge, compounded by their frequent coexistence and the current absence of a validated, non-invasive imaging modality capable of specifically quantifying mural fibrosis in CD [41]. In mural fibrosis, collagen fibers deposit in the bowel wall, involving at least the mucosal and submucosal layers. Fibrotic strictures are noticeable by persistent luminal narrowing and wall thickening, without the signal characteristics associated with inflammation. In contrast, Inflammatory activity typically presents as restricted diffusion on imaging, T2 hyperintensity and mural hyperenhancement [42]. Discriminating between reversible inflammatory and permanent fibrotic components is clinically critical yet difficult, particularly in the context of active inflammation occurring concurrently with chronic fibrosis. Cine MRI provides a unique assessment of intestinal motility, offering functional insights that complement conventional morphological evaluations and establishing its validity as a biomarker for disease activity [20,30,31]. Prior studies have established that intestinal motility is significantly reduced within strictured segments compared to non-stenotic bowel [29]. Extending this finding, Van Rijn et al. [43] and Rimola et al. [44] have further elucidated that the altered motility patterns in the bowel proximal to a stricture are directly associated with the presentation of clinical symptoms.

Patients with higher motility respond better to treatment is as increased motility suggests less smooth muscle hypertrophy and less fibrosis in the affected bowel segments [45,46]. Fibrosis can result in rigid and non-compliant bowel segments, thereby diminishing their capacity to respond dynamically to inflammation and medical interventions [45,46,47]. Furthermore, severe and chronic inflammation can lead to permanent impairment of the autonomic nervous system, resulting in dysmotility and a loss of normal bowel function [48]. This preservation of motor function potentially indicates a milder disease state, where the retention of normal bowel physiology facilitates a more robust therapeutic response to anti-inflammatory agents, thus improving the prognosis for averting disease advancement. One study reported that patients exhibiting preserved or enhanced motility are likely to experience less structural and functional damage, which may render their bowel more responsive to the anti-inflammatory effects of anti-TNF therapy [49] Another study demonstrates that increased mean motility scores in intestinal strictures are significantly associated with favourable treatment outcomes [40]. This supports the prognostic value of motility metrics, which reflect underlying bowel function, for predicting a positive response to anti-TNF therapy in pediatric and young adult patients with structuring CD.

## 7. Discussion

Cine MRI’s primary strength is its ability to quantify small bowel motility non-invasively, providing essential insights into disease mechanisms. Most studies in this review were performed on adults. Consistent evidence indicates that active CD is characterized by significantly decreased motility in inflamed segments, with inverse correlations observed between motility indices and established inflammation biomarkers (e.g., fC and CRP), endoscopic scores (CDEIS), and histopathologic activity (eAIS) [20,29,33]. Mechanistically, this decreased motility results from inflammatory infiltration, neural dysfunction, and fibrotic remodeling [28], which cine MRI uniquely captures through dynamic visualization of peristalsis. Preliminary studies, such as that by Beek et al. [34], report promising accuracy (AUC: 0.93) for this differentiation based on pre-stenotic motility; however, these findings originate from small, single-center cohorts and require validation in larger studies. Furthermore, the lack of a standardized histopathological reference standard for fibrosis remains a significant challenge in validating these imaging biomarkers. Recent 3D methods improve this further by measuring local velocities along intestinal centerlines, achieving near-perfect separation of motile versus non-motile segments (AUC: 0.97; van Harten et al. [35]).

This technique also shows promise as an early biomarker for therapy response; however, most of these studies were conducted among adults. In adults, anti-TNFα responders experience a significant improvement in motility (median increase: 73.4%) within 12 weeks, which is strongly associated with clinical improvement and normalization of CRP levels [31]. Pediatric studies also note an increase in motility as early as 6 weeks after starting biologics [30]. This functional recovery often occurs before visible changes on conventional MRI, indicating that cine MRI could allow for earlier treatment adjustments. However, the MOTILITY trial tempers expectations: while cine MRI detects active inflammation with high Sn (71%), it lacks prognostic value for 1-year remission and cannot reliably guide long-term biologic decisions [32]. Improvements in motility can be observed as early as 12 weeks following the initiation of anti-TNFα therapy, indicating that these enhancements may serve as an early marker for treatment response. Thus, it complements but does not replace current biomarkers used for therapy monitoring. The disparity between studies on motility MRI in CD reflects its distinct roles in short-term monitoring versus long-term prediction. Earlier research, such as Plumb et al. (2015) [31] and Dillman et al. (2022) [30], established it as a highly sensitive tool for confirming early biological responses to therapy, functioning as a “thermometer” for current inflammatory activity. An increase in motility shortly after treatment initiation robustly indicates the drug is having a positive effect. However, the large MOTILITY trial demonstrated that this early signal does not translate into reliable long-term prognostication. It cannot predict who will maintain remission a year later, as it cannot account for subsequent factors like immunogenicity or disease phenotype evolution. In practice, this means motility MRI is valuable for confirming early treatment efficacy but should not be used to make definitive long-term decisions about therapy.

The pragmatic algorithm for incorporating cine MRI into the management of CD effectively outlines its utility across various clinical scenarios, including baseline assessments, suspected strictures, and equivocal inflammation, as well as for evaluating treatment response at 6–12 weeks. However, it is essential to recognize its limitations, particularly in cases of fixed strictures with no distention or poor bowel preparation, as these conditions can significantly limit its diagnostic value. For reporting findings, adhering to a minimum dataset that includes patient information, imaging details, motility assessments, and recommendations is crucial for ensuring clarity and facilitating subsequent clinical decisions. By providing a structured approach, it can enhance the integration of cine MRI into routine practice while effectively addressing its limitations and strengths.

Several studies have shown that the GIQuant software provides an objective score that can be used as a proxy for disease activity. As intestinal inflammation increases, motility decreases, resulting in a lower GIQuant score [20,30,36,38]. Some studies have shown a correlation between the GIQuant motility score and the validated MRI index, MaRIA [20,36,38]. However, most of these studies reported that the GIQuant motility scores may not replace endoscopy for diagnosing CD, but they can be used to monitor how therapy affects small bowel motility and inflammation [29,30].

It is essential to consider these advances within their current limitations; cine MRI should be viewed as a valuable complement to, rather than a replacement for, traditional MRE sequences and endoscopic evaluation. A major hurdle to widespread clinical use of quantitative cine MRI is the lack of standardized, validated motility thresholds. The studies reviewed here use a variety of metrics and proprietary software (e.g., GIQuant), and were performed on different patient groups and scanner platforms. Furthermore, in these studies, Cine MRI sequence parameters varied slightly based on patient anatomy, system (1.5 T vs. 3 T) and scanner vendor. As a result, the reported thresholds are experimental and cannot be directly applied to clinical practice. Future research should focus on conducting large, multi-center studies aimed at establishing and validating universally accepted cutoff values for bowel motility that are linked to clinically meaningful outcomes. This approach is crucial for improving the diagnostic value of motility tests in clinical settings and ensuring that results can be reliably used across diverse patient populations.

A major obstacle to the broad clinical use of cine MRI is the significant lack of standardization in technical parameters. Current research shows substantial differences in field strength (1.5 T versus 3 T), the use of proprietary versus open-source post-processing software (e.g., GIQuant, in-house developed code), and how the motility metric is defined (e.g., motility index, displacement, frequency). This technical variability greatly hampers the reproducibility of results between studies and centers, preventing the establishment of a universal, clinically useful cutoff value to differentiate inflammation from fibrosis. Large-scale, multicenter studies are urgently needed in the future, emphasizing harmonized acquisition protocols to develop validated, standard metrics crucial for integrating into clinical guidelines.

## 8. Future Directions

The widespread adoption of cine MRI as a primary CD biomarker depends on standardizing protocols and overcoming important methodological challenges identified over the past decade. Future research should specifically address the issue of circularity (motility versus morphology) by focusing on motility metrics obtained from non-inflamed or mildly affected segments, providing independent evidence that functional impairment exists beyond visible structural disease. Establishing standardized methods to reduce spectrum bias is crucial; this involves conducting prospective, multi-center studies with strict enrollment protocols that ensure a complete, continuous spectrum of disease activity is represented, moving away from simple active/inactive categories. Furthermore, therapeutic monitoring requires tight adherence to the reference standard; researchers must strictly follow a ≤7-day window between MRE and the endoscopic reference standard to prevent confounding effects of natural disease progression. This precision is vital to validate the use of motility as a tool for repeated assessments and clinical response monitoring. Finally, while initial work, such as the pediatric feasibility study by Cococcioni et al. [36] established the value of per-segment analysis (for localized diagnostic correlation), the field must develop a standardized, clinically relevant per-patient motility score (for evaluating overall disease burden and therapeutic response) to aid in systemic management and inform overall therapeutic decisions.

## 9. Conclusions

Cine MRI has advanced from a research tool to a clinically practical method for assessing CD severity, identifying strictures, and detecting early treatment responses. Its incorporation into routine MRE protocols enhances functional assessment without extending scan times. Future advancements in quantification and standardization are needed to fully unlock its potential. For the foreseeable future, its role remains adjunctive to conventional MRI and clinical evaluation, but it holds promise for adding a functional component to personalized treatment strategies in CD.

## Figures and Tables

**Table 1:** Advantages and limitations of cine MRI in inflammatory bowel disease.

Pros	Cons
No ionizing radiation and no need for intravenous contrast; provides unique functional data complementary to anatomical MRI; valuable for repeated assessments;demonstrated utility in both adult and pediatric populations.	Requires advanced post-processing software (e.g., GIQuant), which may not be universally available; Increase acquisition and analysis time; moderate inter-observer variability;lack of standardized acquisition protocols and diagnostic thresholds; sensitivity to motion artifacts (though less so with free-breathing techniques).

**Table 2:** Summary of studies that use cine MRI in the diagnosis of CD.

Population No. Study Type Scanner Ref.	Aim	Reference Standard	Acquisition	Findings
Adults				
CD:53 Adults Prospective 1.5 T or 3 T [29]	Evaluated the correlation between cine MRI quantified small bowel motility, patient symptom burden, and inflammatory activity	fC for inflammatory burden; HBI for subjective symptom burden	Cine, breath-hold MRE; Sequence: TrueFISP (1.5 T) or BTFE (3 T) with temporal resolution: 1 image/s over a 20 s breath hold	A significant negative correlation was found between global motility variance and fc (rho = −0.33, *p* = 0.015). A highly significant negative correlation was found between global motility variance and total HBI (rho = −0.45, *p* < 0.001). For each 0.01 unit reduction in global motility variance, the odds of a patient reporting a worse HBI well-being score increased by 0.19 (95% CI, 0.06−0.50; *p* < 0.001). Se, Sp, or AUC not reported.
CD: 82 Adults Prospective/Multi-institutional study 3 T [20]	Assessed the association between the terminal ileal motility and CD activity assessed with both the histopathologic score (eAIS) and endoscopic severity index (CDEIS)	Endoscopic CDEIS Histopathologic eAIS	Cine, breath-hold MRE Sequence (2D coronal TrueFISP/BTFE) acquired during a 22 s breath hold. The temporal resolution of the dynamic images was 1.1 s per section with a section thickness of 10 mm focused on the terminal ileum.	Reduced motility (0.30 au) was highly sensitive for both endoscopically and histologically (92% and 92%, respectively) defined active inflammation, but had moderate Sp (61% and 71%, respectively). MaRIA had 75% Sn and 74% Sp. Terminal ileal motility had a negative correlation with CDEIS (r = −0.59; 95% confidence interval [CI]: 0.7, −0.4) and eAIS (r = −0.61; 95% CI: 0.7, −0.5). TI motility: 0.86, 0.87 (against CDEIS and eAIS, respectively). Terminal ileal motility, evaluated by an automatic algorithm, has a higher Sn and similar specificity to MaRIA.
CD: 59 Adults Retrospective 1.5 T and 3 T [27]	To verify the association between intestinal inflammation and reduced motility within the study participants	CD: 59 Adults Retrospective	Coronal cine 2D breath steady state free precession (SSFP) FIESTA (Fast Imaging Employing Steady State Acquisition)/True FISP (Fast Imaging with Steady Precession) sequences and the cine temporal resolution was 1 s per image	A significant association was observed between the presence of a stricture and reduced small bowel motility (OR 0.40, 95% CI 0.17–0.95, *p* = 0.038). The study reported that in on cine MRI sequences, small bowel strictures in CD patients are associated with decreased small bowel motility.
CD:134 HC: 22 Adults Prospective 1.5 T [33]	Assessed value of motility indices derived from MRE images as a biomarker for CD in clinical practice	Signs of inflammation on MRE, endoscopy, and/or elevated lab markers (e.g., CRP, fC)	Free-breathing coronal TrueFISP images were acquired	Motility index of terminal ileum (active CD vs. HCs): AUC: 0.736 (95% CI: 0.625–0.848), *p* = 0.002. while (active CD vs. inactive CD): AUC: 0.682 (95% CI: 0.579–0.785), *p* = 0.001. In patients with active CD, the motility index in the terminal ileum and ileum was significantly lower compared to HCs and inversely correlated with mural thickness in the terminal ileum. However, there were no differences in the motility index between HCs and inactive CD. Motility index has poor to acceptable discrimination ability for CD activity.
CD:10 HC: 14 Adults Prospective 3 T [35]	To develop a technique for quantifying and characterizing small intestinal motility in 3D cine MRI, to differentiate motile, non-motile, and peristaltic motion patterns	Consensus of two blinded raters	3D coronal dynamic balanced fast field echo (bFFE) during a breath hold	The mean (range) AUC of 0.81 (0.783–0.819) for the detection of peristalsis and AUC across all folds was 0.97 (0.975–0.979) for the detection of <50% motile segments. Absence of motility was significantly more common in CD compared to HC. Discernible peristalsis was significantly more common in HC compared to CD. The mean absolute velocity of intestinal content was significantly lower in CD (median [IQR] 1.06 [0.61, 1.56] mm/s) compared to HCs (median [IQR] 1.84 [1.37, 2.43] mm/s) (*p* < 0.001).
CD: 28 adults Prospective 3 T [34]	To evaluate the feasibility of distinguishing inflammatory (i.e., inflammatory and mixed) strictures from chronic (i.e., non-inflammatory) strictures in stricturing CD through the use of cine MRI	Histopathology	Coronal dynamic single slice 2D bFFE cine MRI (20 s breath-hold)	AUC for chronic stricture detection (based on pre-stricture dilatation motility): 0.93 (95% CI, 0.78–1.0, *p* = 0.011). No difference in motility was found between strictures. Motility in the pre-stricture dilatation was significantly higher for chronic (non-inflammatory) strictures (median: 289.5 AU [IQR 188.0–362.9]) compared to inflammatory strictures (median: 113.1 AU [IQR 83.6–142.4]) (*p* = 0.004). Quantified motility of pre-stricture dilatations showed high accuracy (AUC 0.93) in distinguishing chronic from inflammatory strictures.
Pediatric				
CD: 25 Pediatric Retrospective 1.5 T and 3 T [36]	Examine the association between quantified terminal ileal motility and fC, Crohn Disease MRI Index (CDMI), histopathological activity grading	Primary: Active disease defined as eAIS > 0; Secondary: Crohn Disease MRI Index (CDMI) and fC levels	Coronal bSSFP	Motility index was higher in normal bowel (median: 0.21) compared to inflamed bowel (median: 0.12) (*p* = 0.02). Motility index has an AUC of 79.2%. Agreement between the two readers was high with an intraclass correlation coefficient of 0.98 (CI 0.95–0.99, *p* < 0.001). A negative correlation was found between disease activity and terminal ileum motility, indicating that bowel motility decreases as intestinal inflammation intensifies. In active diseases, the motility index decreases and can be a diagnostic marker with medium accuracy.

**Table 3:** Summary of some studies that used cine MRI in the treatment of adult CD.

Study, Population, Follow-Up, and Ref.	Reference Standard for Response	Acquisition and Motility Metric	Threshold for Prediction	Outcomes (Se, Sp, AUC, and OR) and Key Findings
CD Adults (*n* = 46) single-center follow-up: Median 55 weeks (retrospective) and after median 12 weeks (prospective) [31]	Primary: Physician global assessment (retro) or ≥3-point HBI drop + no surgery/switch (Prospect). Secondary: CRP normalization, MaRIA < 11.	Acquisition: Coronal Cine (TrueFISP/BTFE), multiple breath-holds. Metric: Change in segmental motility. Scanner: 1.5 T or 3 T.	Any increase in motility from baseline to follow-up.	For Anti-TNFα response: • Se: 93.1% (95% CI: 78.0–98.1%); • Sp: 76.5% (95% CI: 52.7–90.4%); • AUC: 91.5% (82.3–100.0%); • OR (per 0.01 AU increase): 1.24 (1.08–1.43); *p* = 0.0027. For CRP normalization: greater motility increase in normalized group (73.4% vs. 5.1%, *p* = 0.0035). For MaRIA < 11: greater motility increase in responders (94.7% vs. 15.2%, *p* = 0.017). Responders to anti-TNFα treatment showed significantly higher improvements in motility (median = 73.4% increase from baseline) compared to non-responders (median = 25% reduction, *p* < 0.001). Good agreement between two readers at both baseline and follow-up Cine MRI for small bowel CD (ICC = 0.65, *p* < 0.001 and ICC = 0.71, *p* < 0.001, respectively). AUROC: change in motility: 0.93
CD adults (*n* = 86) prospective, multicenter follow-up: 1 year (Visit 3) [32]	Endoscopic response (≥50% drop in SES-CD or London Index) at 1 year.	Acquisition: Cine MRI. Metric: GIQuant score change (stable/improved). Scanner: 1.5 T or greater.	Stable or improved motility from baseline (V1) to 12–30 weeks (V2).	Cine MRI vs. CRP for predicting 1-yr response: • Se: 71.0% (52.0–85.8%) vs. CRP 45.2%; • Sp: 30.9% (19.1–44.8%) vs. CRP 67.3%; • AUC: 0.48 vs. CRP 0.53 (*p* = 0.65). Variations in cine MRI, fC, and CRP were unable to predict remission or response reliably at 1 year. Stable bowel motility assessed through cine MRI had high Sn but lower Sp for indicating response or remission. Moderate negative correlation between cine MRI scores and disease activity as quantified by morphological MRE parameters.
CD adults (*n* = 86) prospective, multicenter (agreement study) follow-up: 1 year (Visit 3) [39]	Endoscopic response (≥50% drop in London Index or SES-CD) at 12 months.	Acquisition: Cine MRI. Metric: GIQuant score. Scanner: 1.5 T or greater.	Not Applicable	Agreement: • Inter-observer ICC: 0.59 (95% CI: 0.51–0.66) to 0.70 (95% CI: 0.61–0.78); • Intra-observer ICC: 0.70 (95% CI: 0.44–0.86) and 0.71 (95% CI: 0.44–0.86).

**Table 4:** Summary of some studies that used cine MRI in the treatment of pediatric CD.

Population, Study Type, Follow-Up, and Ref.	Reference Standard for Response	Acquisition and Motility Metric	Threshold for Prediction	Outcomes (Se, Sp, AUC, and OR) and Main Findings
CD pediatric/young adult (*n* = 35); prospective, controlled. Follow-up: baseline, 6 weeks, and 6 months post-treatment. [30]	Diagnosis: CD vs. HCs. Response: Stringent composite clinical/biological remission at 6 months.	Acquisition: Cine MRI. Metric: GIQuant score (FDA-cleared). Scanner: 1.5 T.	For diagnosis (CD vs. HC): normalized score ≤ 0.46.	For diagnosis (CD vs. HC): • Se: 80.0% (95% CI: 56.3–94.3%); • Sp: 93.3% (95% CI: 68.1–99.8%); • AUC: 0.88 (95% CI: 0.72–0.96). For predicting 6-month remission: • Neither baseline nor 6-week change predicted remission (AUC ~0.50–0.71, *p* > 0.05). • Cine MRI motility increased significantly with treatment (*p* = 0.03–0.04). • Cine MRI motility correlated negatively with CRP (r = −0.30 to −0.33), ESR (r = −0.35) and wPCDAI (r = −0.36 to −0.43). Excellent: ICC = 0.89 (95% CI: 0.83–0.93). CD patients showed increases in intestinal motility between baseline and 6 weeks after initiation of biologic treatment, while the entire study cohort showed increases between baseline and 6 months after treatment initiation. However, intestinal motility scores in CD patients did not significantly change between six weeks and six months following the start of treatment.
CD Pediatric/young adult (*n* = 40); retrospective. Follow-up: 6 months post-treatment. [40]	Response was defined as sustained symptom improvement with or without imaging improvement, without escalation of medical therapy, with need for discontinuation of anti-TNF agents within 6 months of initiating or adjusting treatment.	Acquisition: Cine MRI. Metric: Mean Motility Score (AU) via GIQuant. Scanner: 1.5 T or 3.0 T.	General: <150 AU (reduced), >300 AU (normal). Analysis: Continuous mean value.	Stricture motility in responders vs. non-responders: Mean: 176.03 ± 128.63 vs. 67.83 ± 33.88 (*p* = 0.006) • AUC: 0.81 (Overall); 0.75 (Subgroup ≤ 21 years); • OR (for mean): 1.020 (1.00–1.04). Estimated standard deviation for stricture motility also exhibited significant variation, being 72.06 ± 55.55 for responders compared to 30.27 ± 25.79 for non-responders (*p* = 0.02). Sn and Sp were not explicitly reported.

## Data Availability

The datasets used and/or analyzed during the current study are available from the corresponding author on reasonable request.

## Supplementary Materials

The following supporting information can be downloaded at: https://www.mdpi.com/article/10.3390/diagnostics15233078/s1, Videos S1 and S2: A 26-year-old female patient with a confirmed diagnosis of Crohn’s disease underwent Cine-MRI (BTFE sequence). Scans were acquired at two timepoints: prior to the initiation of treatment (baseline, left) and following a 6-month therapeutic intervention (follow-up, right). These videos are courtesy from the MOTILITY trial data.
