# Supplementary material for: The Evolving Role of Cine MRI in Crohn’s Disease: From Functional Motility Analysis to Precision Management: A Review of the Last 10 Years
Source: Diagnostics (Basel). 2025 Dec 3;15(23):3078. doi: 10.3390/diagnostics15233078 (PMC12691373; doi:10.3390/diagnostics15233078)
Supplement: Supplementary file 1 [file diagnostics-15-03078-s001.zip › diagnostics-3978235-supplementary.pptx]

## Slide 1
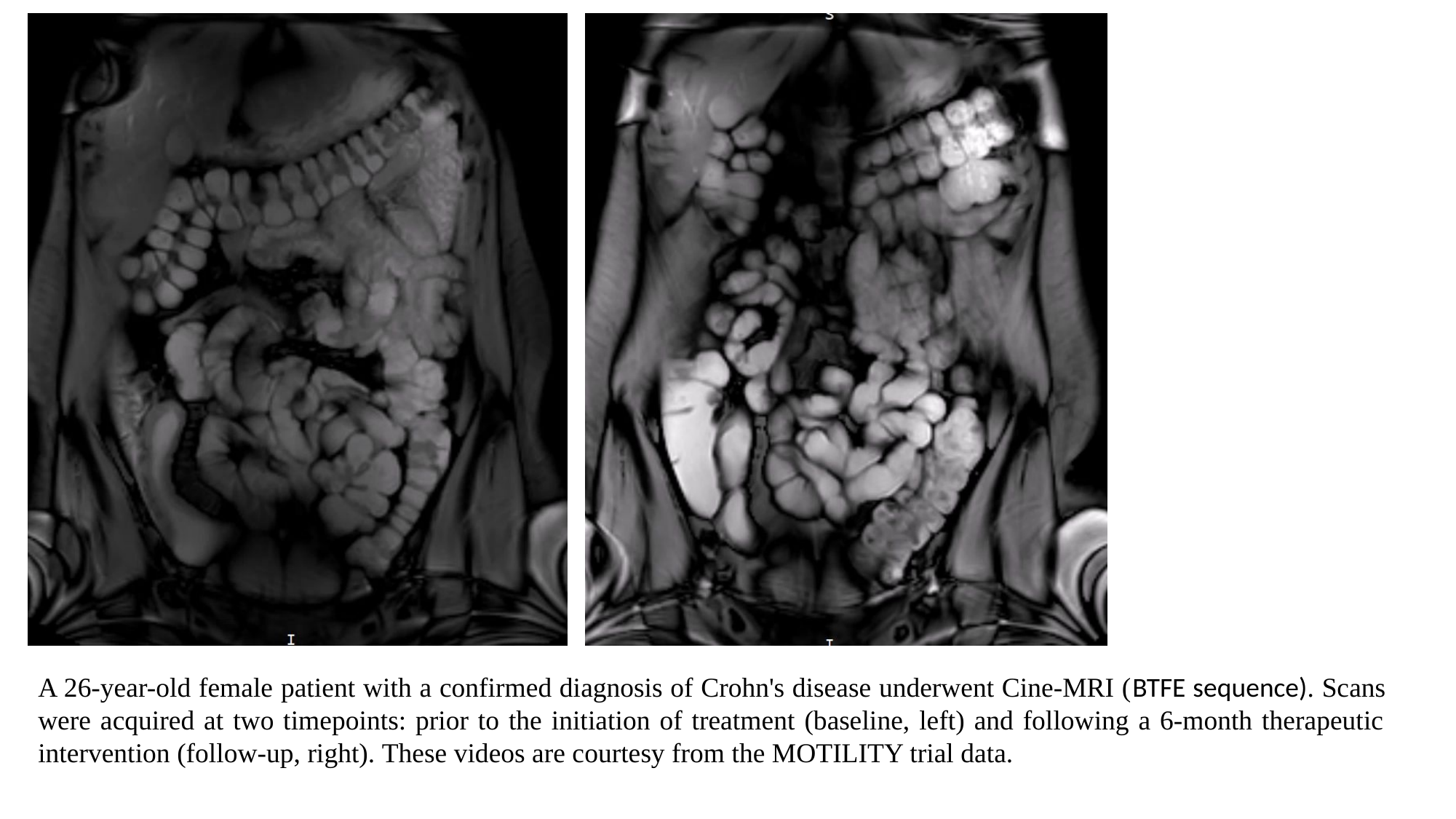

A 26-year-old female patient with a confirmed diagnosis of Crohn's disease underwent Cine-MRI (BTFE sequence). Scans were acquired at two timepoints: prior to the initiation of treatment (baseline, left) and following a 6-month therapeutic intervention (follow-up, right). These videos are courtesy from the MOTILITY trial data.
